# Developing a Staging Scheme for Essential Tremor: A Discussion of Organizing Principles

**DOI:** 10.5334/tohm.812

**Published:** 2023-11-07

**Authors:** Abhishek Lenka, Elan D. Louis

**Affiliations:** 1 Department of Neurology, Baylor College of Medicine, Houston, TX, USA; 2 Department of Neurology, University of Texas Southwestern, Dallas, TX, USA

**Keywords:** Essential Tremor, Staging, Classification, Clinical, Prognosis

## Abstract

Essential tremor (ET) is a chronic, progressive neurological disease that may negatively affect patients’ lives. While there has been considerable progress in ET research, some fundamental issues remain unaddressed. One such issue is *disease staging*. Staging schemes have inherent value and are part of the dialogue that clinicians have with other movement disorders patients. We highlight the value of and challenges with developing a staging system for ET and organize a discussion around the potential steps in developing such a system. Diseases for which there are staging schemes generally have a number of shared characteristics. ET has numerous features that would lend themselves to a staging scheme: emerging evidence supporting the existence of a premotor phase of disease, insidious onset, progressive worsening of arm tremor, spread of tremor to other body regions, the observation that patients seem to be at increased risk for other conditions within the same organ (i.e., emergence of Parkinson’s disease and Alzheimer’s disease in excessive numbers of ET patients), pathological changes in the cerebellum whose evolution can be ordered from (i) those that compromise the physical integrity and physiological function of Purkinje cells, (ii) subsequent changes that are reparative and regenerative, and (iii) eventual cell death. Challenges to formulating a staging scheme are the absence of both a biological marker and an “end stage” of disease. The sum of combined evidence suggests that a staging scheme would be of value. We provide initial thoughts as to how to begin to structure such a staging scheme.

## Introduction

Essential tremor (ET) is a chronic, progressive neurological disease; its prevalence in the population is high, making it one of the most common movement disorders among adults [1]. Recent evidence from clinical, neuroimaging, and postmortem studies suggest that the disease could be neurodegenerative [2,3,4,5,6,7,8,9,10,11,12,13,14,15,16,17,18]. Although in the past mislabeled as “benign” [19], the impact of ET on patients’ as well as caregivers’ lives can be significant [20,21,22,23,24,25,26,27], and this reinforces the importance of research that advances the field. While there has been considerable progress in ET research over the past decade, several fundamental issues have not been addressed, and these merit the attention of scholars in this field. One such issue is *disease staging*. In clinical settings, ET patients inquire about how advanced their disease is relative to that of other individuals and what they can expect moving forward, yet no staging system exists for ET, placing clinicians in a situation in which they can only provide somewhat ambiguous and imprecise responses, which are not grounded within a standardized framework. Staging schemes have been developed and are part of the dialogue that clinicians have with patients who have other movement disorders (e.g., Parkinson’s disease [PD], Huntington’s disease [HD]) [28,29], but not at this point for ET. The underlying question is whether we would benefit from a staging system for ET, and if yes, what our approach should be in developing such a staging scheme.

In this article, we discuss the potential value as well as the challenges with developing a staging system for ET and elucidate the potential steps to develop such a system. Our goal is to begin to frame discussion, encourage continued work, and ultimately, advance the field.

## Why is staging important?

**Figure 1:**
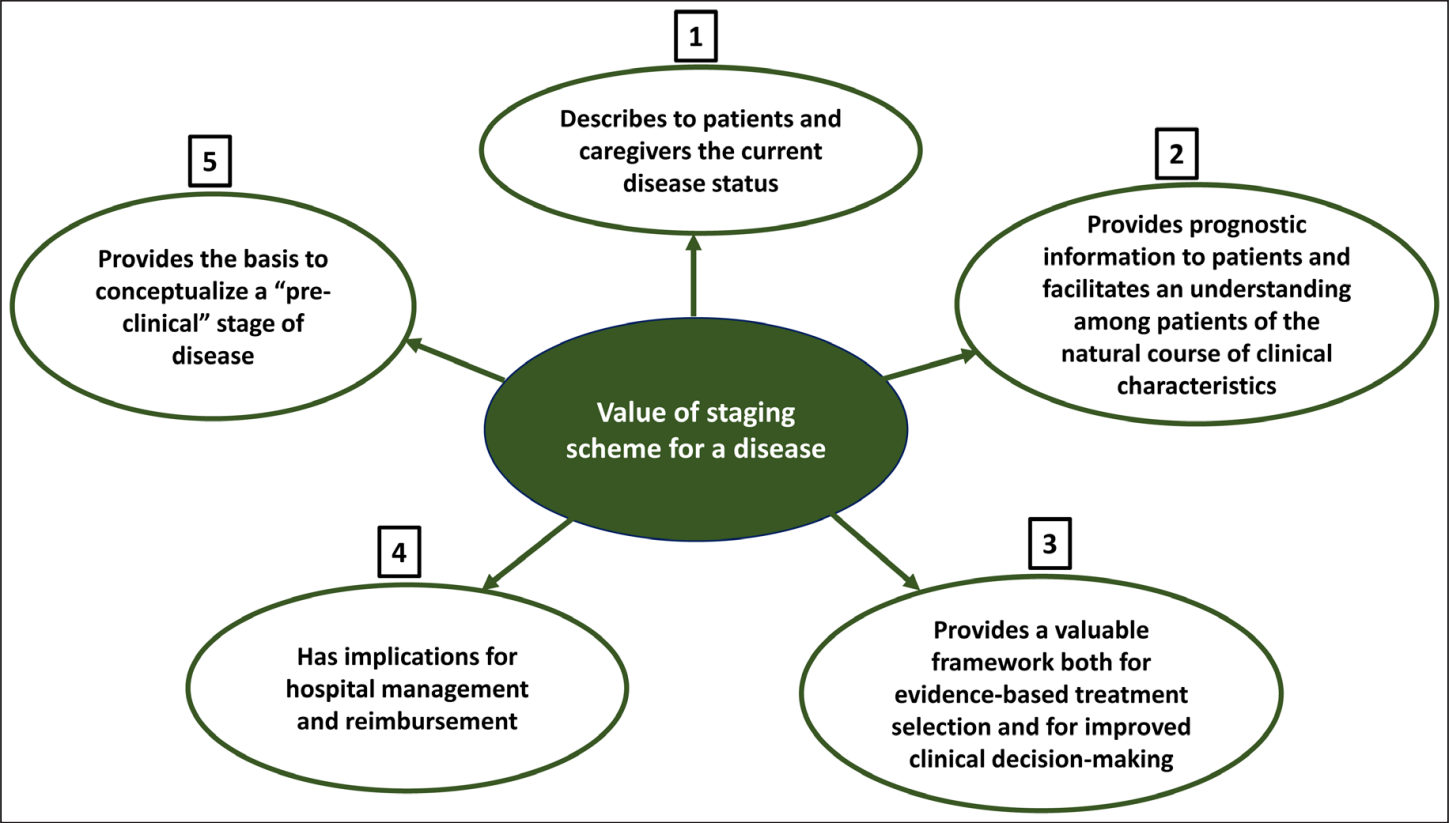
The value of a staging scheme for a disease.

The concept of “grading” and “staging” of disease originated in the field of oncology to provide a uniform description of the index of aggressiveness (grade) and anatomical extent (stage) of cancers [30]. Grading and staging have played a crucial role in cancer, not only for the treating physicians, but also for the patients and caregivers. Over the years, the concept of staging has also been implemented in numerous non-cancerous conditions. A few among the many examples are chronic kidney disease (CKD) [31] and alcoholic liver disease [32], and in the neurological sphere, PD [28] and Alzheimer’s disease (AD) [33]. Additionally, an integrated staging system was recently proposed for HD [29]. While “grading” may not be relevant in neurodegenerative disorders, “staging” has significant value (Figure 1).

One of the immediate benefits of staging is that it describes to patients and caregivers the current disease status. For example, when a neurologist describes a PD patient as having a Hoehn and Yahr (H&Y) stage-1, this clearly depicts a patient with unilateral parkinsonism without balance impairment [28]. Similarly, stage-3 HD depicts patients with loss of functional capacity, in contrast to stage-2 HD, which depicts a patient with symptoms and signs of HD without loss of functional capacity [29].

Staging can provide prognostic information to patients, thereby providing a roadmap of what lies ahead - a general guide about how abilities may change over time. As such, staging facilitates an understanding among patients of the natural course of clinical characteristics. In sum, staging defines discrete anchor points in the course of a disease, which are clinically detectable, reflect severity, and possess clinical significance for prognosis and choice of therapeutic modality [34].

Clear communication regarding staging also results in a shared framework among healthcare providers. Additionally, with well-documented staging, treatment can be tailored to patient needs, further optimizing patient care. In this way, staging provides a valuable framework both for evidence-based treatment selection and for improved clinical decision-making during patient management [35].

Furthermore, disease staging has implications for hospital management and reimbursement. In this way, staging is important for the purposes of calculating hospital reimbursement, as treatment of more advanced disease is associated with greater intensity of required services, higher costs and additional resources [36].

The presence of a staging scheme also provides the basis to conceptualize a “pre-clinical” stage of disease. Disease-modifying or disease halting interventions at this early stage are likely to be more effective than when such interventions are initiated only after the disease has become symptomatic [37,38].

## What are characteristics of diseases for which there are associated staging schemes?

Diseases for which there are staging schemes generally share a number of characteristics. To begin with, these diseases often begin insidiously. At this early point, clinical or pathological features are often difficult or even impossible to clearly distinguish from those found in a normal state. This stage, which lies somewhere between normal and abnormal, i) may be endpoint itself, ii) may be transient, with patients reverting back to the normal state, or iii) may be a transitional precursor to the disease state. An example of such a stage is atypical endometrial hyperplasia, a precancerous lesion of endometrial carcinoma [39]. Similarly, mild cognitive impairment is seen as a pivotal or transitional state that i) can be an endpoint itself, ii) might revert to a normal cognitive state, or iii) is a precursor to dementia [40,41].

Along a similar but not identical vein, there may be a pre-clinical stage of disease. That is, a point at which the underlying disease has been set in motion and pathophysiological processes have commenced on some level, but before appreciable hallmark features have made their appearance. In infectious diseases, this concept has long been established. More recently, with respect to neurodegenerative diseases such as PD, a variety of features (e.g., rapid eye movement sleep disorder, constipation) are regarded as markers of pre-clinical disease [42,43,44].

As a corollary, in neurodegenerative disorders that have core motor features, including PD and HD, cognitive and psychiatric features can predate the onset of motor symptoms and formal disease diagnosis. A number of studies have demonstrated the depression and anxiety may pre-date the onset of motor symptoms in PD [42,45,46]. Similarly, in HD, cognitive deficits may be robust indicators of the underlying disease process, prior to reaching criteria for a motor diagnosis of HD [47].

Diseases that are progressive, both in terms of clinical features as well as underlying biological changes, are the ones that are amenable to staging. Often, these changes can be identified and carefully tracked over time, with staging intervals assigned to certain points in the evolution of features.

On the other end of the disease spectrum, patients often reach an “end stage” in which spread of disease has made it more pervasive, and this is often associated with extreme disability and death. In the case of AD, for example, patients become completely dependent on caregivers, being unable to independently eat or move without assistance. The disease becomes so severe that patients may become bedridden. Complications such as immobility, swallowing disorders, and malnutrition can lead to death [48,49].

Some diseases may increase the risk of developing additional diseases within the same organ and, as such, this development represents a downstream result of the initial disease. As such, the initial condition serves as a risk factor for the second condition. Patients with the initial disease, therefore, enter a stage of illness in which they begin to express additional conditions. For example, both hepatitis C virus and fatty liver disease increase the risk of developing hepatocellular carcinoma [50,51]. The mechanisms for the observed relationship are not clear, although shared genetic and epigenetic factors might underlie both conditions, or alternatively, the pathophysiological cascade resulting from the first condition (i.e., free radical formation, inflammation, cell death) may give rise to the second condition.

In addition to *clinical* features that lend themselves to a *clinical* staging scheme, staging schemes can also be built around *pathological* features or the combination of both. As such, an identifiable cascade of pathological changes is one of the key hallmarks of diseases that lend themselves to staging schemes.

## Formulating a staging scheme for ET: arguments in favor of

As a disease, ET has numerous features that would lend themselves to the development of a staging scheme. We review these below.

A characteristic of diseases that are staged is that they have a “pre-clinical” stage. More specifically, in degenerative disorders of the motor systems, non-motor features, including cognitive, psychiatric and others, can predate the onset of motor symptoms and a formal disease diagnosis. In the section above, we referred to examples both in PD and HD. With respect to ET, longitudinal data provide emerging evidence of what may be a premotor phase of disease involving cognitive and/or psychiatric manifestations [52,53,54,55]. In an epidemiological study in Spain, among unaffected individuals who eventually developed incident ET during follow up, premotor evaluations revealed greater cognitive dysfunction and a faster rate of cognitive decline than seen in individuals who did not develop ET [53]. In the same cohort, such premotor evaluations have also revealed more self-reported depression, antidepressant medication use, and shorter sleep duration in individuals who eventually developed incident ET when compared with controls [54,55]. In cross-sectional studies, the presence of certain personality traits in ET cases also suggest the existence of a set of definable characteristics that predate motor features [52]. Additional cohort studies would serve to further validate and understand this putatitve stage of disease.

Another characteristic of such diseases is that they begin insidiously. In ET, the tremor begins nearly imperceptibly. On neurological examination, it can be difficult for neurologists to distinguish physiological tremor, a normal condition, from early stage ET, thereby calling on electrophysiological testing to provide additional information [56]. A result of this subtle beginning is that patients often have difficulty recalling their precise age of onset. In one prospective, longitudinal study, 125 ET cases were asked at different time intervals to report their age of onset; in 20 – 25% of cases, the responses at different time intervals differed by as much as 10 years [57,58]. In some reportedly “unaffected” individuals, head tremor is the presenting feature of ET, beginning as a transient, subtle head wobble only brought out under certain conditions; this is not appreciated by the individual him/herself [59]. Similarly, in a family study of ET, of the eight relatives diagnosed by a study neurologist as having mild, early ET, 5 (62.5%) were asymptomatic (i.e., the patients did not report that they had tremor or ET) [60].

As mentioned above diseases that are progressive, both in terms of clinical features as well as underlying biological changes, are the ones amenable to staging. Data from numerous studies serve to document the progressive nature of ET. Longitudinal investigations have consistently reported a gradual increase in tremor severity over time. For instance, a clinical series of 128 consecutive patients with ET who were followed for routine care, reported an annual increase of 12% in upper limb tremor severity [61]. A prospective, longitudinal research study that captured ET patients from non-clinical settings, reported a progressive worsening in upper limb tremor scores such that the average annual increase in tremor severity from baseline was estimated to be between 3.1% and 5.3% and the median annual increase from baseline was between 1.8% and 2.0% [62]. A study on 116 ET patients demonstrated progressive worsening tremor on Archimedes spirals [63]. A retrospective study of 50 patients with ET, using the Glass scale, reported a progressive worsening of tremor and a significant negative correlation between age at the onset of tremor and the rate of progression [64]. A cross-sectional study that stratified 335 ET cases by decade of disease duration, noted that with increases in disease decade, there was progressively more severe and more widespread tremor [65]. Not only do objective metrics of tremor showcase this progression, but prospective, longitudinal studies have similarly revealed that the majority of ET patients self-report worsening of tremor over time [66].

Tremor in ET can be phenomenologically complex, with what starts as a simple kinetic tremor eventually evolving into a tremor that may have a postural component, an intentional component and a rest component [67]. A number of these features correlate with disease duration, such that longer standing ET cases are more likely to develop these features [68,69].

The scope of progression in ET extends beyond the severity of arm tremor, encompassing the spread of tremor to other body regions. Cross sectional data show that with longer disease duration, the prevalence of cranial tremors increases; for example, a study of 335 ET cases reported that among those with duration <10 years, 31.3% had voice tremor, compared to 48.6% among those with tremor duration ≥40 years [65]. In addition, 2.1% of the former had tremor in the head, jaw, and the voice vs. 8.2% of the latter [65]. A study of 37 outpatients evaluated at two time intervals approximately 3 years apart, noted an increase in the proportion who exhibited head and voice tremors at the second time interval [70]. Aside from motor features, studies have highlighted that a subset of patients with ET develop non-motor features, and one of the commonly reported non-motor features is cognitive impairment, with rates of conversion to dementia being higher than that seen in the general population [71].

Based on the data above, ET seems to be a progressive disease that may entail worsening upper limb tremor severity, development of tremor during an increasing variety of activation conditions, the expansion of tremor to different body regions and the potential progressive development of non-motor impairments. The term “ET-plus”, proposed in a recent viewpoint paper [72], but not widely accepted [73], likely represents a more advanced stage of the disease in which a host of motor and non-motor features are evident in ET patients, as would be expected based on the above [74].

As discussed above, diseases may also increase the risk of developing additional diseases within in the same organ, representing in some senses, a downstream effect of the initial disease. As such, the baseline condition is a risk factor for the second condition. Patients with the initial disease enter a stage of illness in which they begin to give rise to these additional diseases. As such, a number of studies have demonstrated that patients with ET are at increased risk of developing incident PD [75,76] as well as incident AD [77,78]. The former association may be due to the preponderance of Lewy pathology found in ET cases [79]; the latter may be due to links between ET and tau pathology [80,81,82]. The emergence of PD and AD in ET patients suggests a potential transition to a more advanced and widespread stage of neurodegeneration.

Above, our discussion was focused exclusively on *clinical* features and *clinical* staging schemes. In addition, staging schemes can also be built around *pathological* features. An identifiable cascade of pathological changes is one of the key hallmarks of diseases that lend themselves to staging schemes. The pathophysiology of ET has not been fully elucidated, although there is mounting postmortem evidence that it is neurodegenerative, with postmortem changes observed primarily in the cerebellar cortex [2,3,11,12,83]. While it is remains unclear as to whether these are the only changes in ET, and what the precise order of evolution of these changes is, some models have been proposed, suggesting a series of early changes that compromise the physical integrity and physiological function of Purkinje cells, subsequent changes that are likely reparative and regenerative, and then eventual cell death [2].

**Figure 2:**
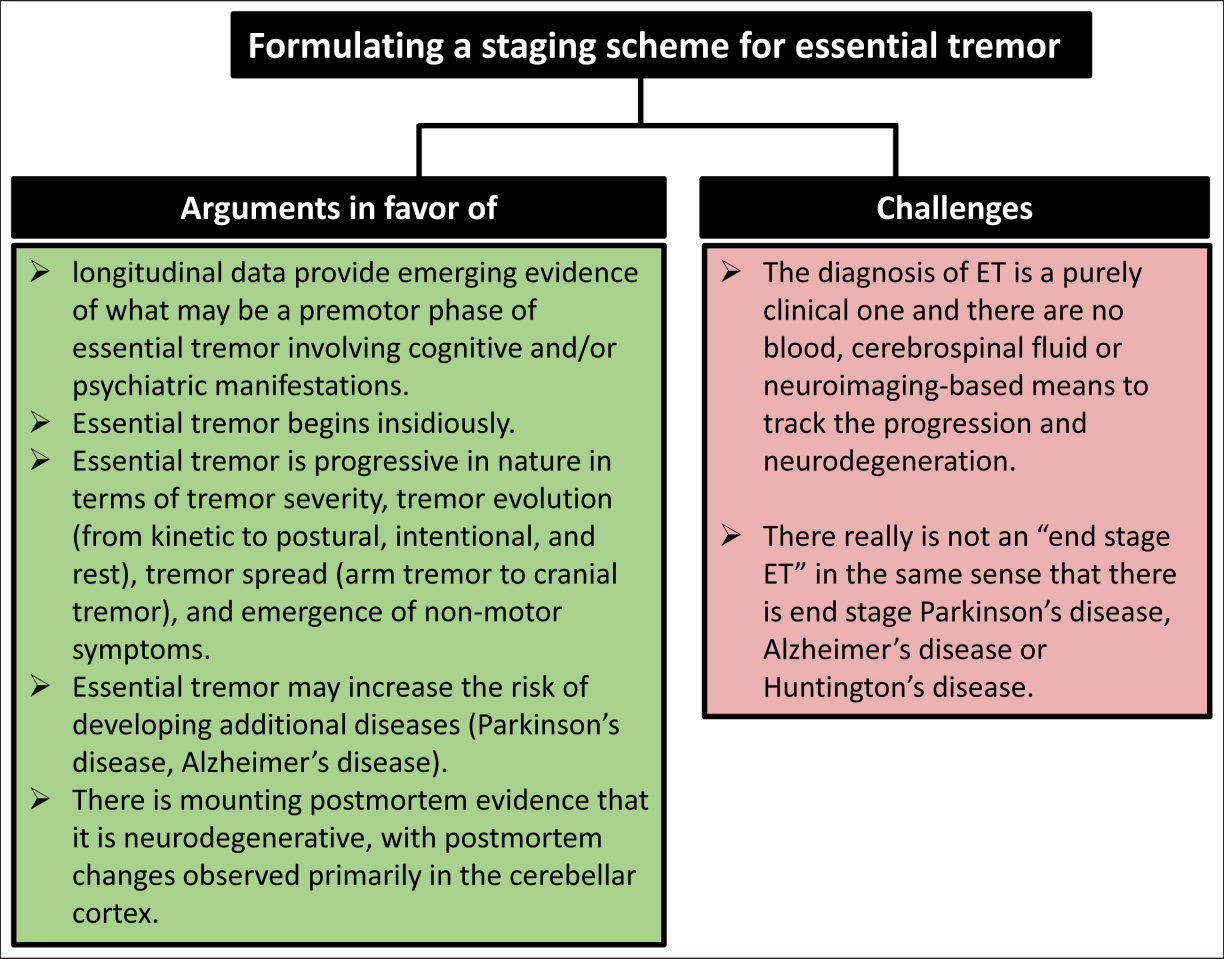
Formulating a staging scheme for essential tremor: Arguments in favor of and challenges.

In summary, in ET, there are numerous features, including both clinical and pathological, that argue for the need of an organizing principle in the form of a staging scheme (Figure 2).

## Formulating a staging secheme for ET: challenges

Unlike numerous neurological diseases for which staging schemes have been developed (e.g., AD, PD, HD), the diagnosis of ET is a purely clinical one and there are no blood, cerebrospinal fluid or neuroimaging-based means to track the progression and neurodegeneration as there is in AD (amyloid positron emission tomography [PET]), in PD (dopamine transporter or fluorodopa PET scan), or HD (magnetic resonance imaging). This would mean that, at least for the moment, an ET staging scheme would not be biomarker-based.

Another challenge is that there really is not an “end stage ET” in the same sense that there is end stage PD, AD or HD. Certainly, ET patients may experience increasing difficulty completing motor tasks and some activities of daily living. In fact, they may not be able to perform some of them at all. However, ET patients do not become bed bound and unable to care for themselves. Although there is an increased risk of mortality in ET [84], the disease itself does not drive patients on a relentless pathway to death. This does not mean that the disease does not progress through certain stages, it just means that the highest stage is not a terminal one.

Finally, the underlying pathology of ET needs to be further elucidated. While numerous of the postmortem changes in ET either track with tremor severity and duration, and a general ordering of postmortem changes has been proposed [2], we are not yet at the point at which a clear staging of postmortem changes has been developed.

Some of the challenges posed above may be one reason why a staging scheme for ET has not been developed to date (Figure 2).

## What would be the components of a staging scheme for ET? initial thoughts

Above, we discussed the practical value for developing a staging scheme for any disease, and we outlined a number of features of ET upon which such a scheme could be based. We also discussed several challenges. In the absence of biomarkers, at present, the clinical staging scheme would center on the presence and evolution of clinical features. Additional biomarker work would open the door for a staging scheme that included two elements – clinical and biomarker-based. Further understanding of the evolution of the observed postmortem changes in the ET cerebellum would be required to develop a parallel postmortem-based staging scheme. Hence, the discussion below focuses on a clinical staging scheme for ET.

In AD and HD, clinical stages divide the disease into mild, moderate, and severe or early, middle and late. It is entirely conceivable that a similar staging scheme could be developed for ET – early/mild, middle/moderate, late/severe. The clinical components of each stage would recognize the progressive nature of ET as well as the features discussed at length above. One major limitation in our knowledge about the evolution of clinical features in ET is that there is a dearth of longitudinal clinical studies, and few prospective, longitudinal studies. This limits our precise understanding of the patterns one sees in the timing of, features of, and extent of changes (e.g., worsening in upper limb tremor, addition of tremors in other activation states [e.g., intention, rest], spread of tremor to cranial and other structures, and development of additional neurodegenerative conditions). More research is needed.

**Figure 3:**
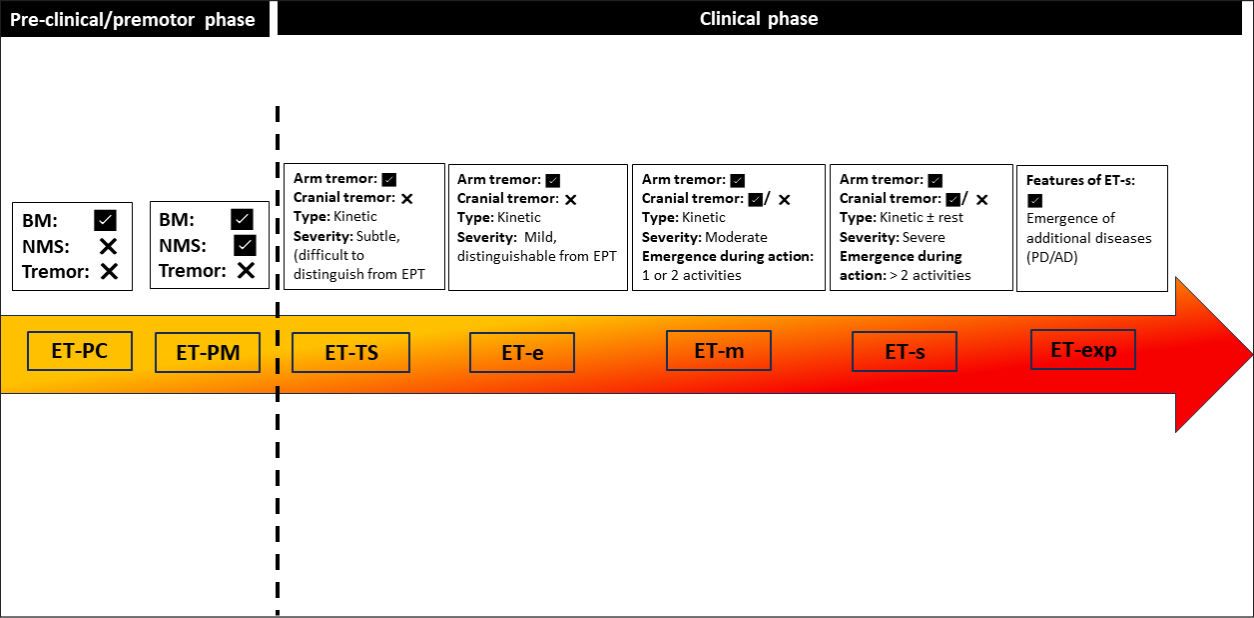
Proposed clinical staging scheme for essential tremor. **BM**: Biomarkers, **EPT**: Enhanced physiologic tremor, **ET:** Essential tremor, **ET-PC:** Preclinical ET, **ET-PM:** Premotor ET, **ET-TS:** ET in transitional stage, **ET-Mi**: Mild ET, **ET-M:** Moderate ET, **ET-S:** Severe ET, **ET-EXP:** ET-expansive disease stage, **NMS:** Non-motor symptoms.

With this information, and that reviewed above, the following clinical staging scheme for ET would seem to be both situationally appropriate and scientifically supported (Figure 3):

1. *Pre-clinical stage (ET-PC)* – disease pathophysiology has commenced, molecular and tissue based changes are evident, but no clinical features are detectable. In the absence of a disease biomarker for ET, at present, this stage is not detectable, and hence, at the moment, this stage remains somewhat theoretic.
2. *Premotor stage (ET-PM)* – Cognitive and/or psychiatric manifestations are evident but abnormal tremor is not evident. There is some evidence for this stage, although more are needed.
3. *Transitional stage (ET-TS)* – tremor is evident, although it is difficult to fully distinguish from physiological or enhanced physiological tremor.
4. *Mild ET (ET-MI)* – mild tremor involving the arms only. Kinetic tremor is evident and distinguishable from physiological or enhanced physiological tremor.
5. *Moderate ET (ET-MO)* - moderate tremor involving the arms. Tremor may have spread to cranial structures. Early emergence of tremor during other activation conditions.
6. *Severe ET (ET-S)* - severe tremor. Tremor may have spread to cranial structures. Emergence of tremor at rest or during several other activation conditions.
7. *Expansive Disease (ET-EXP)* – severe ET with the layering in of other related neurodegenerative conditions (PD and AD)

Several issues merit additional comment. First, although mild, transient isolated head tremor has been reported in relatives of ET cases, current diagnostic schemes do not classify isolated head tremor as ET [59,72,85]. If future work were to substantiate the concept that patients with isolated head tremor have the beginnings of ET, the staging scheme would need to be modified. Similar comments may be made about isolated voice tremor. Second, the scheme we propose is a staging scheme. A grading scheme, were it to be proposed, would attempt to organize patterns in rate of disease progression. Grading and staging schemes, when used synchronously, can provide robust information regarding disease progression and outcomes. Although there is literature assessing rate of progression in ET with respect to specific demographic and clinical features [61], the number of such studies is small, and additional data is needed before an effective grading scheme could be established. Third, the severity of tremor (mild/moderate/severe) may be measured by standardized scales such as Fahn-Tolosa-Marin tremor rating scale or the ET rating assessment scale (TETRAS) [86]. What remains to be determined is the best cut-off scores for “mild,” “moderate,” and “severe” ET.

## Conclusion

A chronic and progressive disease such as ET, with its substantial psychosocial and functional burden on patients’ lives, would benefit from having a well-defined staging scheme. Numerous characteristics of ET support the feasibility of formulating such a system, although there are challenges as well. The staging scheme, for now, would need to be a clinical one. We have identified core elements of such a staging scheme. As researchers and clinicians venture into this endeavor, it is crucial to approach the task with an evidence-based approach first and foremost. The ultimate goal would be to create a clinically relevant and beneficial staging system that goes beyond an academic exercise.
